# PlexinB1 Promotes Nuclear Translocation of the Glucocorticoid Receptor

**DOI:** 10.3390/cells9010003

**Published:** 2019-12-18

**Authors:** Magali Williamson, Ritu Garg, Claire M. Wells

**Affiliations:** School of Cancer and Pharmaceutical Sciences, Faculty of Life Sciences and Medicine, New Hunts House, Guys Campus, King’s College London, London SE1 1UL, UK; ritu.garg@kcl.ac.uk (R.G.); claire.wells@kcl.ac.uk (C.M.W.)

**Keywords:** Plexin, semaphorin, prostate cancer, glucocorticoid receptor, nucleocytoplasmic trafficking, dexamethasone, nuclear localization signal

## Abstract

Androgen receptor (AR) and glucocorticoid receptor (GR) are nuclear receptors whose function depends on their entry into the nucleus where they activate transcription of an overlapping set of genes. Both AR and GR have a role in resistance to androgen deprivation therapy (ADT), the mainstay of treatment for late stage prostate cancer. PlexinB1, a receptor for semaphorins, has been implicated in various cancers including prostate cancer and has a role in resistance to ADT. We show here that activation of PlexinB1 by Sema4D and Sema3C results in translocation of endogenous GR to the nucleus in prostate cancer cells, and that this effect is dependent on PlexinB1 expression. Sema4D/Sema3C promotes the translocation of GR-GFP to the nucleus and mutation of the nuclear localization sequence (NLS1) of GR abrogates this response. These findings implicate the importin α/β system in the Sema4D/Sema3C-mediated nuclear import of GR. Knockdown of PlexinB1 in prostate cancer cells decreases the levels of glucocorticoid-responsive gene products and antagonizes the decrease in cell motility and cell area of prostate cancer cells upon dexamethasone treatment, demonstrating the functional significance of these findings. These results show that PlexinB1 activation has a role in the trafficking and activation of the nuclear receptor GR and thus may have a role in resistance to androgen deprivation therapy in late stage prostate cancer.

## 1. Introduction

Prostate cancer is one of the leading causes of cancer-related deaths in men. The growth of most prostate cancers is initially dependent on signaling via the androgen receptor (AR) and the standard systemic treatment for late stage prostate cancer, androgen deprivation therapy (ADT), involves the blocking of AR signaling by reducing the levels of circulating androgens by chemical means. While very effective at first, development of resistance to ADT usually ensues after 2–3 yrs resulting in lethal castration-resistant prostate cancer (CRPC) [1]. Resistance to ADT occurs by various means including amplification of the AR gene and AR mutation leading to androgen-independent AR signaling and by processes in which AR signaling is bypassed [2].

One of the mechanisms by which prostate tumors acquire resistance to ADT is via upregulation of the glucocorticoid receptor (GR, NR3C1) [3,4,5], the receptor to which the steroid hormone cortisol and other glucocorticoids bind [6]. GR is structurally similar to AR and both are nuclear receptors which bind their respective ligands in the cytoplasm, dimerise and translocate to the nucleus where they bind to specific sites in the DNA and activate transcription of large overlapping sets of genes [6]. The main function of both AR and GR is therefore dependent on translocation of the receptors to the nucleus. AR represses GR expression [3]; consequently blockade of AR signaling, for example by the antiandrogen enzalutamide, leads to an increase in GR expression. An overlap exists between AR and GR target genes [7] and so the increase in GR expression upon ADT leads to activation of a subset of AR target genes by GR, resulting in resistance to ADT. The use of glucorticoids such as dexamethasone to alleviate symptoms of prostate cancer treatment may exacerbate the problem. New methods to block both AR and GR signaling may therefore be required to prevent the development of resistance to ADT [8].

Plexins are a family of cell surface receptors for the extracellular secreted or membrane bound proteins, semaphorins. Ligand binding to PlexinB1 results in activation of the receptor tyrosine kinases ErbB2 [9], EGFR [10], and Met [11] and drives invasiveness of prostate cancer cells [12,13] through regulation of several small GTPases, including Rho [14], R-Ras [15], and Rap [16]. PlexinB1 and its ligands, Sema4D and Sema3C, are associated with tumor progression and bad prognosis of many tumor types [17], including breast [18,19], ovarian [19,20], glioma [21], and melanoma [22] as well as prostate cancer [10,23,24]. PlexinB1, Sema4D, and Sema3C are overexpressed in prostate tumors [10,23,25,26,27] and expression of Sema3C is an independent predictor of biochemical recurrence of prostate cancer [28]. Furthermore, treatment of tumor-bearing mice with an inhibitor of PlexinB1 delays castrate-resistant regrowth of tumors [10], indicating a role for PlexinB1 in resistance to ADT. 

We have recently found that activation of the semaphorin receptor PlexinB1 enhances translocation of AR to the nucleus under low androgen levels [29] and so may contribute to resistance to androgen blockade by increasing the trafficking of AR to the nucleus. The structure and functional similarity of AR and GR and their shared role in resistance to ADT lead us to question whether PlexinB1 may affect nuclear trafficking of GR in addition to that of AR.

## 2. Materials and Methods

### 2.1. Cell Culture 

Prostate cancer cell lines DU145, PC3, and 22Rv1 were grown in RPMI with 10%FCS, Hela cells were grown DMEM with 10% FCS. All cell lines were from ATCC (LGC standards, Middlesex, UK) and were STR typed to confirm their identity.

### 2.2. Antibodies

The following antibodies were used: Mouse anti-GILZ (TSC22D3) monoclonal antibody (Abnova clone 3A5 H00001831-M01, (Abnova, Taipei, Taiwan), goat anti-FKBP51 (Novus af4094, Novus, Centennial, CO, USA), mouse anti-GR (PP-H8004-00 Perseus Proteomics Inc, Tokyo, Japan,), rabbit anti-GR (CST 3660, CST, Danvers, MA, USA), mouse anti-β-actin (ab6276, Abcam, Cambridge, UK), rabbit anti-PlexinB1 (ECM Biosciences, Versailles, KY, USA), anti-lamin (L1293, Sigma, Dorset, UK), and anti-AR (Millipore, Burlington, MA, USA).

### 2.3. Plasmids 

pEGFP GR was a gift from Alice Wong (Addgene plasmid # 47504; http://n2t.net/addgene:47504; RRID:Addgene_47504). The NLS1 site of pEGFP GR was mutated to disrupt NLS1 (IVM mutagenesis kit, NEB, Ipswich, MA, USA) using the primers: TTGTTGTTTGTTTTT CGAGCTTCCAG and CATAAAAGGAATTCAGCAGGCCACTAC to change amino acids Lys[494], Lys[495], Lys[496] to Gln[494], Gln[495], Gln[496].

### 2.4. Immunocytochemistry: Endogenous GR and AR localization

Serum starved PC3 or DU145 cells plated on coverslips were treated with PBS, Sema4D-Fc, Sema3C-Fc (R&D, Abingdon, UK, 2 μg/mL), or dexamethasone (Sigma, 10 nM) for 60 min. Cells were then fixed (4% paraformaldehyde), permeabilized (0.2% triton), stained by immunofluorescence with mouse anti-GR(CST) followed by anti-mouse Alexa Fluor488 (Life Technologies, Carlsbad, CA, USA), phalloidin-TRITC (Sigma) and DAPI. Serum starved 22Rv1 cells were treated with PBS, Sema4D (2 μg/mL) or dihydrotestosterone (DHT, Sigma, 1 nM) for 60 min. Cells were then fixed (4% paraformaldehyde), permeabilized (0.2% triton), stained by immunofluorescence with mouse anti-AR followed by anti-rabbit Alexa Fluor488 (Life Technologies), phalloidin-TRITC (Sigma) and DAPI. Images were taken at x63 magnification using a Zeiss LSM510 confocal microscope and the intensity of staining of Alexa488 in the cytoplasm and nucleus was measured using ImageJ using DAPI staining to outline the nucleus and actin staining to identify the cytoplasm. The ratio of nuclear to cytoplasmic staining for each cell was calculated. (*n* = 3, A minimum of 44 cells were scored per treatment).

### 2.5. GR Localization following Knockdown of PlexinB1 Expression

PC3 cells transfected with non-silencing siRNA or two different siRNAs to PlexinB1 and treated with 10 nM dexamethasone were fixed and stained for GR and the nuclear/cytoplasmic intensity ratio of GR staining recorded as above. (*n* = 3, A minimum of 218 cells counted per treatment).

### 2.6. GR-GFP Subcellular Localization

Cells were transfected with GR-GFP, or GR-NLS1^m^-GFP using Lipofectamine (Invitrogen, Carlsbad, CA, USA) and serum-starved cells were treated with PBS, Sema4D, Sema3C (2 μg/mL) or dexamethasone (10 nM) for 60 min. The cells were fixed, permeabilized (as above), stained by immunofluorescence with phalloidin-TRITC (Sigma) and DAPI. Cells were scored ‘blind’ as to their treatment. Transfected cells were scored according to the following criteria: (a) Intensity of cytoplasmic staining exceeded that of nuclear staining (C > N), (b) Intensity of cytoplasmic staining was equal to that of nuclear staining (*n* = C), (c) Intensity of nuclear staining exceeded that of cytoplasmic staining (N > C). Slides were scored on a Nikon Eclipse Ti spinning disc confocal microscope at 60x magnification. (GR-GFP *n* = 4, A minimum of 259 cells counted per treatment, GR-NLS1^m^-GFP *n* = 4, 182+ cells counted per treatment).

### 2.7. Subcellular Protein Fractionation

Serum-starved PC3 and DU145 cells were treated with PBS or Sema4D-Fc (2 μg/mL) or dexamethasone (10 nM) for 60 min and protein extracted from cytoplasmic and nuclear fractions (subcellular protein fractionation kit, Thermo Scientific) according to manufacturers’ instructions. The subcellular localization of GR was analyzed by immunoblotting using GR (PPinc) and lamin (Sigma) antibodies.

### 2.8. siRNA

PlexinB1 expression was knocked down using two different siRNAs against PlexinB1 (siGenome Dharmacon, Lafayette, CO, USA), (25 nM) and siGENOME non-targetting siRNA pool (Dharmacon) as control using Dharmafect for the transfection according to manufacturers’ instructions. Following transfection, cells were grown for 72 h in RPMI with 10%FCS, which contains corticosteroids allowing activation of GR. Protein levels of FKBP5, GILZ, GR, and PlexinB1 were detected by immunoblotting 72 h after transfection.

### 2.9. Cell Motility

PC3 cells were transfected with non-silencing siRNA (NS) or siRNA to PlexinB1. Transwell migration assays were performed using 24-well, 0.8 μm transwell chambers (BD Biosciences, Berkshire, UK) coated with fibronectin on the lower side. Serum-starved cells (2 × 10^4^ per insert) were placed in the upper chamber with or without dexamethasone (10 nM) and RPMI with 20% FCS in the lower chamber. After 6 hr, cells on the underside were fixed, stained with crystal violet and counted (*n* = 3).

### 2.10. Cell Area Measurement

PC3 cells plated on coverslips were transfected with non-silencing siRNA (NS) or siRNA to PlexinB1. After 72 h, the cells were treated with dexamethasone (10 nM) for 30 min, fixed and stained for GR (anti-GR (CST), anti-rabbit Alexa Fluor 488 (Life Technologies)), actin (actin stain 555, Cytoskeleton Inc., Denver, CO, USA) and DAPI. Cell area was calculated using ImageJ (*n* = 3). A minimum of 141 cells were analyzed per condition.

## 3. Results

### 3.1. Sema4D/Sema3C-PlexinB1 Signaling Increases the Levels of Endogenous Glucocorticoid Receptor in the Nucleus

To determine if activation of PlexinB1 affects translocation of GR to the nucleus we monitored the subcellular localization of GR following treatment with two ligands of PlexinB1, Sema4D and Sema3C, using the AR negative prostate cancer cell lines, PC3 and DU145, as models of androgen-independent prostate cancer.

**Figure 1:**
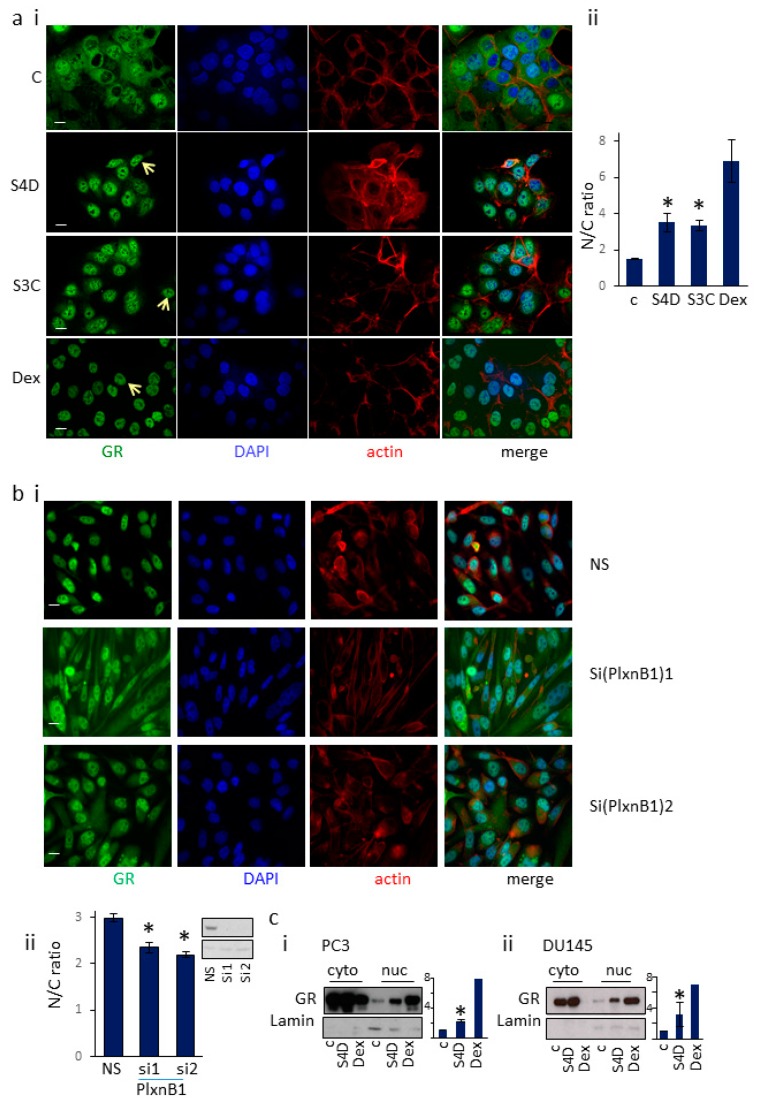
PlexinB1 activation promotes translocation of endogenous GR to the nucleus. (**a**). Stimulation of DU145 cells with Sema4D and Sema3C enhances nuclear translocation of endogenous GR. (i). Representative immunofluorescence images of serum-starved DU145 cells treated with Sema4D (2 μg/mL), Sema3C (2 μg/mL), dexamethasone (10 nM) or vehicle for 60 min, fixed and then stained for endogenous GR (mouse anti-GR, anti-mouse Alexa488), actin (phalloidin-TRITC) or DNA (DAPI) by immunofluorescence. Arrow indicates nuclear GR. (ii). The intensity of staining for GR in the cytoplasm (C) and nucleus (N) was scored and the ratio of cytoplasmic to nuclear staining calculated (*n* = 3), a total of 187+ cells were scored per treatment. Error bars denote SEM, * *p* < 0.05, Ttest; scale bar = 10 μm. (**b**). Knockdown of PlexinB1 expression reduces dexamethasone-induced translocation of GR to the nucleus. PC3 cells transfected with non-silencing siRNA (NS) or two different siRNAs to PlexinB1 and treated with 10nM dexamethasone were fixed and stained for GR and the nuclear/cytoplasmic intensity ratio of GR staining recorded. (i). representative images of PC3 cells transfected with siRNAs indicated and stained for endogenous GR (mouse anti-GR, anti-mouse Alexa488), actin (phalloidin-TRITC) or DNA (DAPI) by immunofluorescence, (ii). Graph of nuclear/cytoplasmic staining intensity ratios for GR in PC3 cells transfected with non-silencing (NS) siRNA or siRNA to PlexinB1. (**c**). Sema4D/PlexinB1 signalling increases levels of endogenous nuclear GR in prostate cancer cells. PC3 (i) or DU145 (ii) cells were serum starved, treated with Sema4D (2 μg/mL), dexamethasone (10 nM) or vehicle and an equal number of cells for each treatment were fractionated into cytoplasmic and nuclear fractions. The presence of GR protein in each fraction was detected by immunoblotting with anti-GR antibody and anti-lamin for nuclear protein. Bar charts depict average band intensity of nuclear fraction (*n* = 3).

Serum starved PC3 or DU145 cells were treated with Sema4D (2 μg/mL), Sema3C (2 μg/mL), dexamethasone (10 nM) (a synthetic GR ligand) or vehicle for 60 min; the cells were then fixed and the localization of GR was detected by immunocytochemistry using a GR-specific antibody and confocal microscopy (Figure 1a, Supplementary Figure S1). Images were scored blind using ImageJ (NIH) and the ratio of nuclear vs cytoplasmic staining was calculated, using DAPI staining to outline the nucleus and actin staining to identify the cytoplasm. DU145 cells treated with Sema4D, Sema3C, or dexamethasone showed significantly higher staining of endogenous GR in the nucleus compared to cells treated with vehicle control (Figure 1a). Similar results were found for PC3 cells (Supplementary Figure S1). Consistent with these results, knockdown of PlexinB1 expression with siRNA significantly decreased the ratio of nuclear/cytoplasmic staining for GR in PC3 cells treated with 10 nM dexamethasone (Figure 1b), indicating that dexamethasone-induced nuclear translocation of GR is in part dependent on PlexinB1 expression.

Cell fractionation studies were next used as a further test of the effect of Sema4D on the translocation of GR to the nucleus. DU145 and PC3 prostate cancer cells (both AR negative and GR positive) were serum-starved and then treated with Sema4D (2 μg/mL), dexamethasone (10 nM) or vehicle for 60 min. Protein extracted from an equal number of cells from each treatment group was then fractionated into cytoplasmic and nuclear fractions and the amount of GR protein in each fraction was measured by immunoblotting. Treatment of PC3 and DU145 cells with Sema4D or dexamethasone resulted in a significant increase in GR levels in the nucleus (Figure 1c). Together these results indicate that activation of PlexinB1 in prostate cancer cells promotes the translocation of endogenous GR from the cytoplasm to the nucleus.

AR is another member of the steroid hormone nuclear receptor family of proteins and has a similar structure to GR. Treatment of 22Rv1 prostate cancer cells with Sema4D or dihydrotestosterone (an AR agonist) resulted in a significant increase in endogenous AR in the nucleus, compared to vehicle control (Supplementary Figure S2).

### 3.2. Sema4D/Sema3C Promotes the Translocation of GR-GFP to the Nucleus

**Figure 2:**
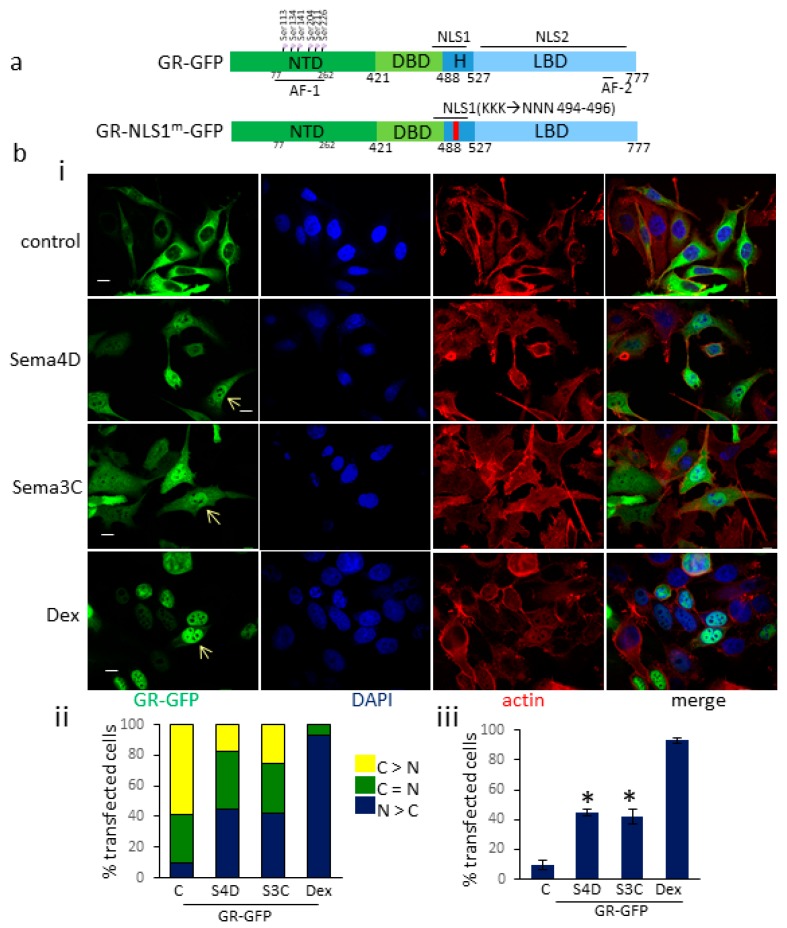
PlexinB1 activation promotes translocation of GR-GFP to the nucleus. (**a**). Diagram to show structure of GR constructs used. NTD: N-terminal domain, DBD: DNA binding domain, H: hinge region, LBD: ligand binding domain, NLS: nuclear localisation sequence. (**b**). (i). Representative images of PC3 cells transfected with GR-GFP and treated with Sema4D (2 μg/mL), Sema3C (2 μg/mL), dexamethasone (10 nM) or vehicle (control) for 60 min. The cells were fixed and stained for actin (phalloidin-TRITC) and DNA (DAPI). Arrow denotes nuclear GR-GFP. Scale bar, 10 μm. (ii). Subcellular localisation of GR-GFP in transfected PC3 cells. Following treatment with Sema4D (2 μg/mL), Sema3C (2 μg/mL), dexamethasone (10 nM) or vehicle (control) for 60 min, PC3 cells transfected with GR-GFP were stained as in (i) and were scored blind and categorised into three groups: (1) nuclear GFP staining > cytoplasmic GFP staining (N > C), (2) nuclear and cytoplasmic GFP staining equal, (*n* = C), (3) cytoplasmic GFP staining > nuclear GFP staining (C > N) and the % of cells in each group scored (*n* = 3). A total of 175+ cells were scored per treatment. (iii) Percentage of cells transfected with GR-GFP in which the intensity of nuclear GFP staining exceeded that of cytoplasmic staining (N > C) following treatment with Sema4D (2 μg/mL), Sema3C (2 μg/mL), dexamethasone (10 nM) or vehicle (control, C) for 60 min (*n* = 3). Error bars denote SEM, * *p* < 0.05, Ttest).

To further investigate the increase in endogenous nuclear GR upon Sema4D and Sema3C treatment, we studied the subcellular localization of GR-GFP transfected into the cells, upon PlexinB1 activation. PC3 cells were transfected with a GR-GFP expression vector encoding all domains of GR (Figure 2a). The transfected cells were treated with Sema4D (2 μg/mL), Sema3C (2 μg/mL), dexamethasone (10 nM) or vehicle. The subcellular localization of the GR-GFP protein in transfected cells was assessed by immunocytochemistry using confocal microscopy. Cells were scored blind and categorized into three groups: (1) Nuclear GFP staining exceeded that of cytoplasmic staining, (2) nuclear and cytoplasmic GFP staining were equal, and (3) cytoplasmic GFP staining exceeded that of nuclear staining. A significant increase in the number of cells in which nuclear GR-GFP staining exceeded that of cytoplasmic staining was observed following treatment with Sema4D compared to vehicle control (Figure 2b). A similar significant increase in nuclear GR-GFP was found following treatment of transfected Hela cells with Sema4D (Supplementary Figure S3).

### 3.3. Sema4D/Sema3C-Induced Translocation of GR-GFP to the Nucleus Requires an Intact NLS1 Site

Two nuclear localization sequence (NLS) domains have been identified in GR [30]. NLS1 facilitates rapid import of GR via the importin-Ran system. NLS1 is formed of a bipartite basic motif and overlaps with the DNA binding domain (DBD). Mutation of the C-terminal cluster of basic amino acids (lysines 494–496) in NLS1 impairs ligand-induced nuclear import of GR and disrupts binding of importin-α to GR [31]. In contrast, NLS2 facilitates import of GR via an importin-independent system. Nuclear import via NLS2 is less efficient and slower than via NLS1, and GR which retains only a functioning NLS2 site is only partially localized to the nucleus [31]. The structure NLS2 is less well characterized and spans most of the ligand binding domain [30].

**Figure 3:**
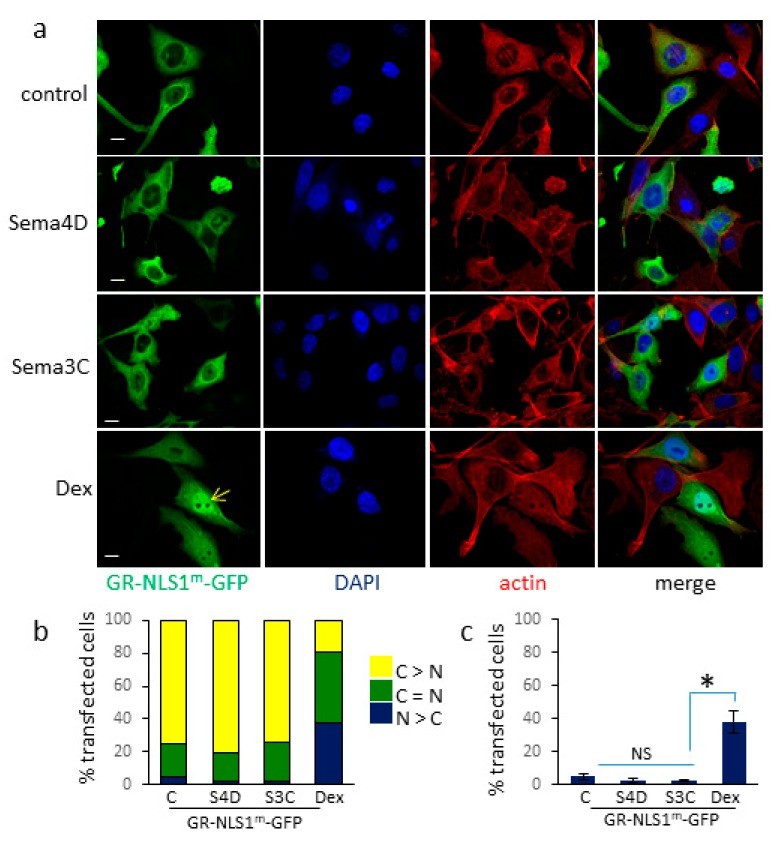
Increase in Sema4D/3C-stimulated nuclear translocation of GR-GFP requires NLS1. (**a**) Representative images of PC3 cells transfected with GR-NLS1^m^-GFP and treated with Sema4D (2 μg/mL), Sema3C (2 μg/mL), dexamethasone (10 nM) or vehicle (control) for 60 min and stained as in Figure 2. Arrow denotes nuclear GR-NLS1^m^-GFP. (**b**) Subcellular localisation of GR-NLS1^m^-GFP in transfected PC3 cells. Following treatment with Sema4D, Sema3C (2 μg/mL), dexamethasone (10 nM) or vehicle (control) for 60 min, PC3 cells transfected with GR-NLS1^m^-GFP and stained and scored as in Figure 2 (*n* = 3). A total of 160+ cells were scored per treatment. (**c**) Percentage of cells transfected with GR-NLS1^m^-GFP in which the intensity of nuclear GFP staining exceeded that of cytoplasmic staining (N > C) following treatment with Sema4D (2 μg/mL), Sema3C (2 μg/mL), dexamethasone (10 nM) or vehicle (control) for 60 min (*n* = 3). Error bars denote SEM, * *p* < 0.05, NS = not significant, Ttest).

In order to investigate the requirement for NLS1 and NLS2 of GR in Sema4D and Sema3C-induced translocation of GR-GFP to the nucleus, cells were transfected with a GR-GFP expression vector in which lysines 494–496 were mutated to glutamine to disrupt the NLS site (GR-NLS1^m^-GFP, Figure 2a). Mutation of NLS1 reduced nuclear localization of GR-GFP upon dexamethasone treatment, as expected. In contrast, mutation of NLS1 completely inhibited Sema4D and Sema3C-induced translocation of GR to the nucleus; levels of nuclear GR in cells treated with Sema4D or Sema3C were comparable to those of cells treated with vehicle control (Figure 3c). Similar results were found in Hela cells transfected with GR-NLS1^m^-GFP (Supplementary Figure S4). These results show that NLS1 is required for Sema4D/Sema3C-induced translocation of GR to the nucleus.

### 3.4. PlexinB1 Affects Protein Levels of Glucocorticoid-Responsive Genes

**Figure 4:**
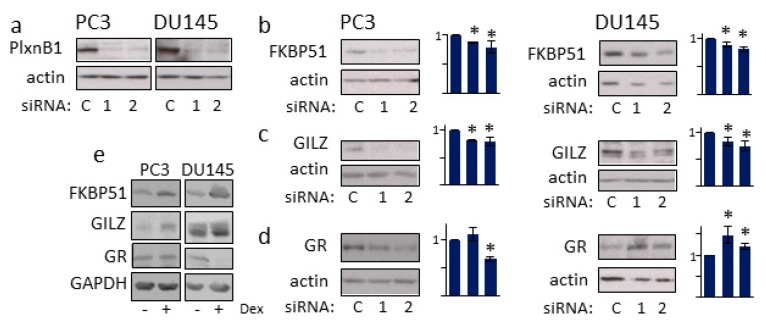
PlexinB1 knockdown affects the level of glucocorticoid-responsive gene products. PC3 and DU145 cells were transfected with two different siRNAs to PlexinB1 or non-silencing siRNA(control) and then grown in medium with 10%FCS (which contains corticosteroids to stimulate GR activation). Protein levels of PlexinB1 (control) (**a**), FKBP51 (**b**), GILZ (**c**) and GR (**d**) were detected by immunoblotting. (**e**) PC3 or DUI45 cells were treated with dexamethasone (10 nM) and protein levels of FKBP51, GILZ, and GR were detected by immunoblotting. Bar charts show band intensities relative to actin (*n* = 3). Error bars denote SEM, * *p* < 0.05, Ttest.

FK506-Binding Protein 5 (FKBP51) and glucocorticoid-induced leucine zipper protein (GLIZ, TSC22D3) genes are GR-target genes whose expression is induced by dexamethasone (Figure 4e) [32,33,34]. To determine if PlexinB1 signaling affects expression of GR-responsive genes, PC3 and DU145 cells were transfected with two different siRNAs against PlexinB1 or control non-silencing siRNA and the levels of FKBP51 and GLIZ protein expression were monitored by immunoblotting. Knockdown of PlexinB1 expression resulted in a significant decline in levels of both FKBP51 and GILZ (Figure 4).

The NR3C1 gene, which encodes GR, has negative glucocorticoid response elements in its promoter and GR expression is regulated by GR in a feedback loop [35]. The effect of PlexinB1 depletion on GR protein levels in the prostate cancer cell lines was cell-context specific, as previously reported [6]: Knockdown of PlexinB1 expression in DU145 cells resulted in an increase in the levels of GR protein with both siRNAs used, while knockdown with one siRNA in PC3 cells resulted in a decrease in GR levels (Figure 4d).

### 3.5. GR Regulates PlexinB1 Protein Levels

**Figure 5:**
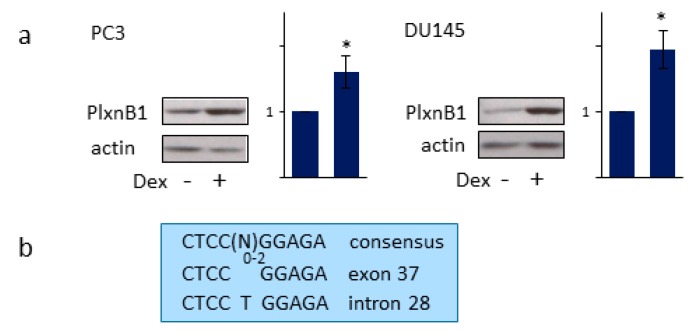
GR activation increases PlexinB1 protein levels. (**a**) PC3 and DU145 cells were treated with dexamethasone (10 nM) or vehicle and the protein levels of PlexinB1 were detected by immunoblotting. Bar charts show band intensities relative to actin (*n* = 3), error bars denote SEM, * *p* < 0.05, Ttest. (**b**) Sequence of the consensus inverted-repeat glucocorticoid binding site (IR-GBS) motifs and 2 possible IR-GBS sites in exon 37 and intron 28 of the PlexinB1 gene.

Treatment of PC3 and DU145 with dexamethasone increased the levels of PlexinB1 protein, as shown by immunoblotting (Figure 5a). Interestingly two inverted-repeat glucocorticoid binding site (IR-GBS) motifs of consensus sequence CTCC(N)_0–2_GGAGA are present in the PLXNB1 gene: CTCCGGAGA in exon 37 and CTCCTGGAGA in intron 28 (Figure 5b). These results suggest that PlexinB1 expression is transcriptionally regulated by GR, although the results are also consistent with a mechanism by which GR activation results in an increase in PlexinB1 protein stability.

### 3.6. Knockdown of PlexinB1 Expression Antagonizes the Reduction in Cell Migration Induced by Dexamethasone

**Figure 6:**
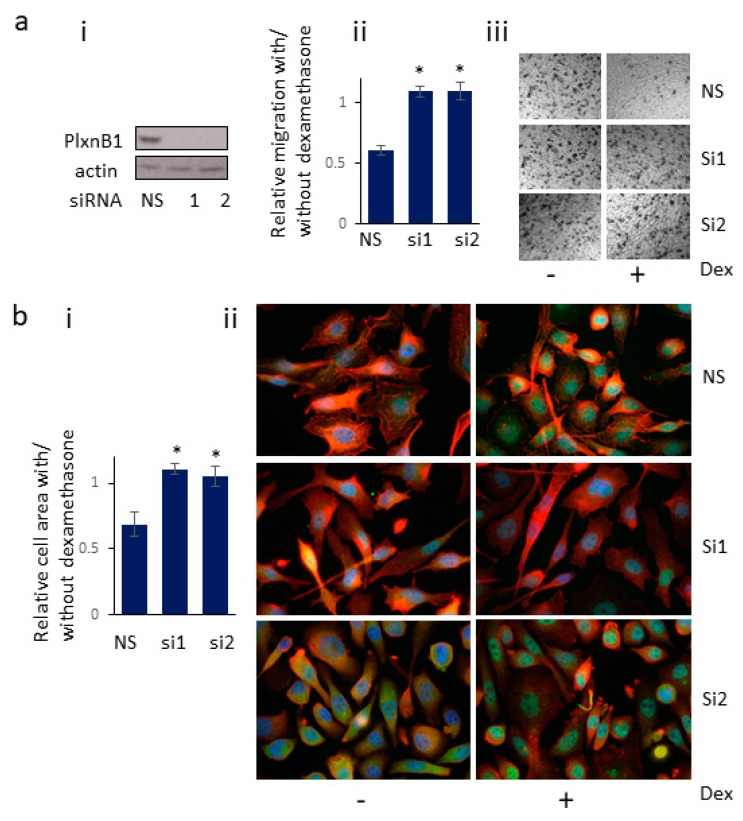
Knockdown of PlexinB1 expression antagonises the reduction in cell migration and cell size induced by dexamethasone. (**a**) Cell migration. (i). PC3 cells were transfected with non-silencing siRNA (NS) or siRNA to PlexinB1 and knockdown of expression detected by immunoblotting (representative image). (ii) Cell motility was assessed in transwell assays in the absence or presence of dexamethasone (10 nM) and the ratio of cells migrated in the presence/absence of dexamethasone (Dex) calculated. Error bars denote SEM, * *p* < 0.05, Ttest (iii). Representative images of migrated cells in transwell assay. (**b**) Cell area. (i). PC3 cells plated on coverslips were transfected with non-silencing siRNA (NS) or siRNA to PlexinB1. After 72 h, the cells were treated with dexamethasone for 30 min, fixed and stained for GR (Alexa488, green), actin (phalloidin 555 red) and DAPI (blue). Cell area was calculated using ImageJ and the ratio of cell area in the presence /absence of dexamethasone calculated. Error bars denote SEM, * *p* < 0.05, Ttest, (ii). Representative images of cells with and without dexamethasone treatment.

Activation of GR with dexamethasone affects cell motility of prostate cancer cells [36]. To determine if this function of GR is dependent on PlexinB1 expression, PlexinB1 expression was knocked down using siRNA to PlexinB1 or non-silencing siRNA and cell migration was assessed in transwell assays. Treatment with 10 nM dexamethasone reduced the cell motility of PC3 cells and knockdown of PlexinB1 expression antagonized this effect (Figure 6a), demonstrating that expression of PlexinB1 is required for the dexamethasone-induced decline in motility of PC3 cells.

### 3.7. Knockdown of PlexinB1 Expression Antagonizes the Dexamethasone-Induced Reduction in Cell Area

PC3 cells were transfected with siRNA to PlexinB1 or non-silencing siRNA as control and then were treated with dexamethasone (10 nM) for 30 min. The fixed cells were stained for actin, GR and DAPI and the cell area measured using ImageJ. Treatment of control cells with dexamethasone resulted in a significant decrease in cells size. In contrast no significant change in cell size was found upon dexamethasone treatment in cells in which PlexinB1 expression had been knocked down (Figure 6b), indicating that dexamethasone-induced cell shrinkage is in part dependent on PlexinB1 expression.

## 4. Discussion

Our results indicate that activation of PlexinB1 enhances the translocation of the glucocorticoid receptor from the cytoplasm to the nucleus, thereby promoting GR function. Plexins activate several receptor tyrosine kinases and RhoGTPases [17] resulting in rapid short-term changes to the cytoskeleton and changes in cell motility. The effect of PlexinB1 activation on nuclear transport may be a mechanism by which extracellular semaphorins also have longer term effects on the responding cell by regulating trafficking of transcription factors and gene expression.

The mechanism by which PlexinB1 signaling affects nuclear localization of GR is not known. GR is reported to contain two nuclear localization sequence (NLSs). NLS1 is positioned near the DNA binding domain/ hinge region of the GR protein and NLS2 overlaps with the ligand binding domain (Figure 2a) [30]. Nuclear import via NLS1 occurs through importin-α/βbinding while NLS2 acts independently of the importin system [31].

Dexamethasone treatment of cells transfected with GR-GFP, which has an intact NLS1 and NLS2 sequence, resulted in nearly total localization of GR in the nucleus. Mutation of NLS1 in GR considerably reduced nuclear translocation of GR induced by dexamethasone, although GR-NLS1^m^-GFP did enter the nucleus in response to dexamethasone and in most transfected cells GR-NLS1^m^-GFP was localized to both cytoplasm and nucleus after one hour. The partial translocation of GR-NLS1^m^-GFP to the nucleus in response to dexamethasone most likely results from the less efficient importin-independent mechanism mediated by NLS2 [30,31].

Sema4D/Sema3C treatment of cells transfected with GR-GFP with an intact NLS1 and NLS2 sequence resulted in nearly 50% localization of GR predominantly in the nucleus, significantly higher than vehicle-treated controls. In contrast to dexamethasone treatment, Sema4D/Sema3C-induced nuclear translocation of GR-GFP was completely abolished upon mutation of NLS1; subcellular localization of GR-NLS1^m^-GFP was similar between Sema4D and vehicle control-treated cells. These results indicate that Sema4D and Sema3C-mediated nuclear translocation of GR is dependent on NLS1 and so is likely to occur through the importin system.

PlexinB1 activation also enhances the nuclear translocation of AR [29], a steroid nuclear receptor of similar structure to GR. AR also relies on importin-based nuclear transport system for nuclear import. Similarly, Yang et al. [37] reported that endogenous *NF-κB* translocates to the nucleus in response to Sema4D in endothelial cells. Together these findings suggest that semaphorin/plexin signaling may have a general role in the regulation of nuclear trafficking of a set of transcription factors.

The levels of PlexinB1 protein increased in response to GR activation suggesting that PlexinB1 is a GR-regulated gene. The increase in PlexinB1 protein levels could result from increased transcription of PLXNB1 or an increase in protein stability. Two consensus inverted-repeat GR-binding sites (IR-GBS) are found in the PlexinB1 gene in exon37 and intron 28. Other steroid receptors such as AR can bind to the canonical GBS sequence, but IR-GBS is specific for GR binding. Further studies are required to confirm whether any of these sites constitute functional glucocorticoid response elements. A functional androgen response element (ARE) is found in intron 2 of the gene for Sema3C, a ligand for PlexinB1, and Sema3C is transcriptionally regulated by AR [38]. Together these results uncover an interplay between AR, GR, and plexin signaling pathways with positive feedback loops between GR and PlexinB1 and AR and PlexinB1: (1) PlexinB1 stimulation promotes nuclear translocation and consequent activation of AR [29] and GR, (2) GR promotes PlexinB1 protein levels, (3) AR promotes Sema3C expression/PlexinB1 activation [38] and (4) AR represses GR expression [3], (Supplementary Figure S5).

PlexinB1 and its ligand Sema3C have been shown to promote resistance to androgen receptor pathway inhibition in prostate cancer treatment. Inhibition of either PlexinB1 or Sema3C significantly delays regrowth of prostate cancer xenografts in mice following castration or enzalutamide treatment [10]. In addition, overexpression of Sema3C increases the regrowth of prostate cancer xenografts following castration [10]. In clinical prostate cancer, high Sema3C expression levels are associated with resistance to ADT in patient derived xenografts [39] and an increase in Sema3C is found in CRPC and bone metastases [10]. Upregulation of GR is also associated with resistance to ADT, probably due to GR activation of a subset of AR-regulated genes [3]. The enhanced translocation of GR and AR to the nucleus upon PlexinB1 activation may explain how PlexinB1 activation promotes resistance to ADT in the experiments described above [3,10].

Taken together, our findings show that Sema4D/PlexinB1 signaling promotes the translocation of the glucocorticoid receptor to the nucleus and thereby enhances GR function. PlexinB1 may thus contribute to the development of resistance to androgen withdrawal in CRPC.

## Supplementary Materials

The following are available online at https://www.mdpi.com/2073-4409/9/1/3/s1, Supplementary Figure S1. PlexinB1 activation promotes translocation of GR to the nucleus; Supplementary Figure S2. Stimulation of 22Rv1 with Sema4D enhances nuclear translocation of endogenous androgen receptor; Supplementary Figure S3. Sema4D increases translocation of GR-GFP to the nucleus in Hela cells; Supplementary Figure S4. Increase in Sema4D-mediated nuclear translocation of GR-GFP in Hela cells requires NLS1; Supplementary Figure S5. Diagram to show interplay between AR, GR and plexin signaling pathways.
