# Supplementary material for: PlexinB1 Promotes Nuclear Translocation of the Glucocorticoid Receptor
Source: Cells. 2019 Dec 18;9(1):3. doi: 10.3390/cells9010003 (PMC7017238; doi:10.3390/cells9010003)
Supplement: Supplementary file 1 [file cells-09-00003-s001.pdf]

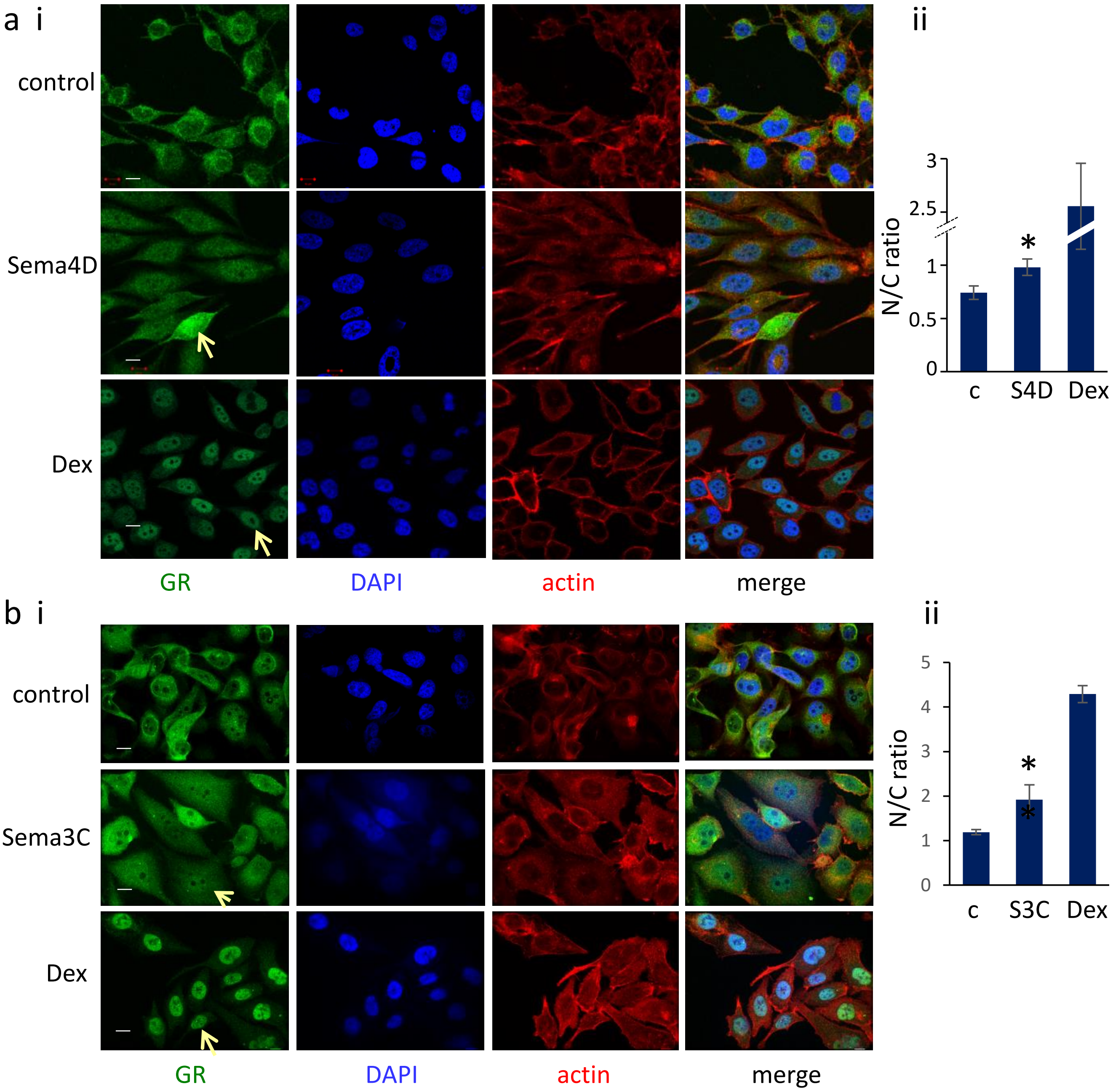

Supplementary Figure 1. PlexinB1 activation promotes translocation of GR to the nucleus

a). Stimulation of PC3 cells with Sema4D enhances nuclear translocation of endogenous GR.

i). Representative immunofluorescence images of serum-starved PC3 cells treated with Sema4D(2ug/ml), dexamethasone (10nM) or vehicle for 60mins, fixed and then stained for endogenous GR (mouse anti-GR, anti-mouse Alexa488), actin (phalloidin-TRITC) or DNA (DAPI) by immunofluorescence. Arrow indicates nuclear GR. ii). The intensity of staining for GR in the cytoplasm(C) and nucleus(N) was scored and the ratio of cytoplasmic to nuclear staining calculated (n=3), a total of 50+ cells were scored per treatment. Error bars denote SEM, * p<0.05, Ttest; scale bar = 10um

b). Stimulation of PC3 cells with Sema3C enhances nuclear translocation of endogenous GR.). Representative immunofluorescence images of serum-starved PC3 cells treated with Sema3C(2ug/ml), dexamethasone (10nM) or vehicle for 60mins, fixed and then stained as in a). Arrow indicates nuclear GR. ii). The intensity of staining for GR in the cytoplasm(C) and nucleus(N) was scored and the ratio of cytoplasmic to nuclear staining calculated (n=3), a total of 50+ cells were scored per treatment. Error bars denote SEM, * p<0.05, Ttest; scale bar = 10um

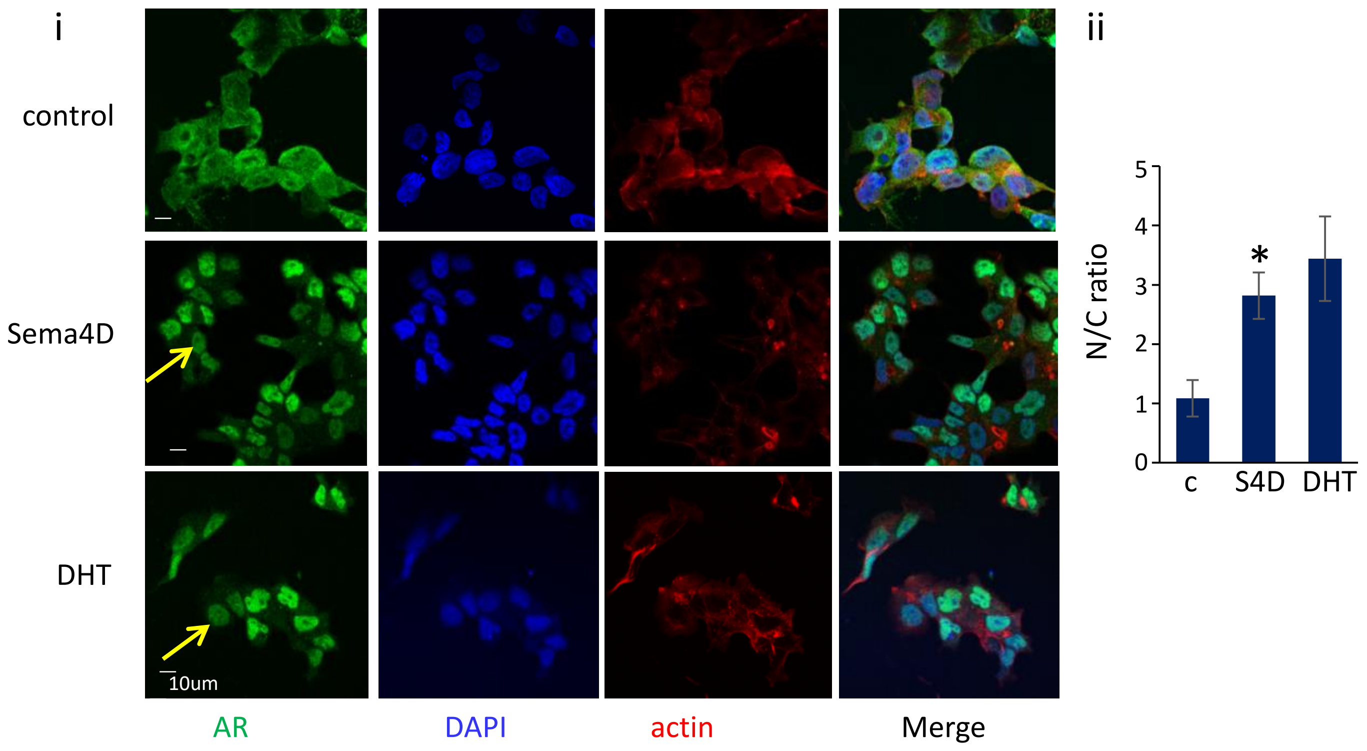

Supplementary Figure 2. Stimulation of 22Rv1 with Sema4D enhances nuclear translocation of endogenous androgen receptor.
i). Representative immunofluorescence images of serum-starved 22Rv1 cells treated with Sema4D(2ug/ml), DHT (1nM) or vehicle for 60mins, fixed with 4% paraformaldyde and then stained for endogenous AR (rabbit anti-AR (anti-rabbit Alexa488), actin (phalloidin-TRITC) or DNA (DAPI) by immunofluorescence. Arrow indicates nuclear AR. ii). The intensity of staining for AR in the cytoplasm(C) and nucleus(N) was scored and the ratio of cytoplasmic to nuclear staining calculated (n=3), a total of 28+ cells were scored per treatment. Error bars denote SEM, * $p<0.05$, Ttest; scale bar = 10um

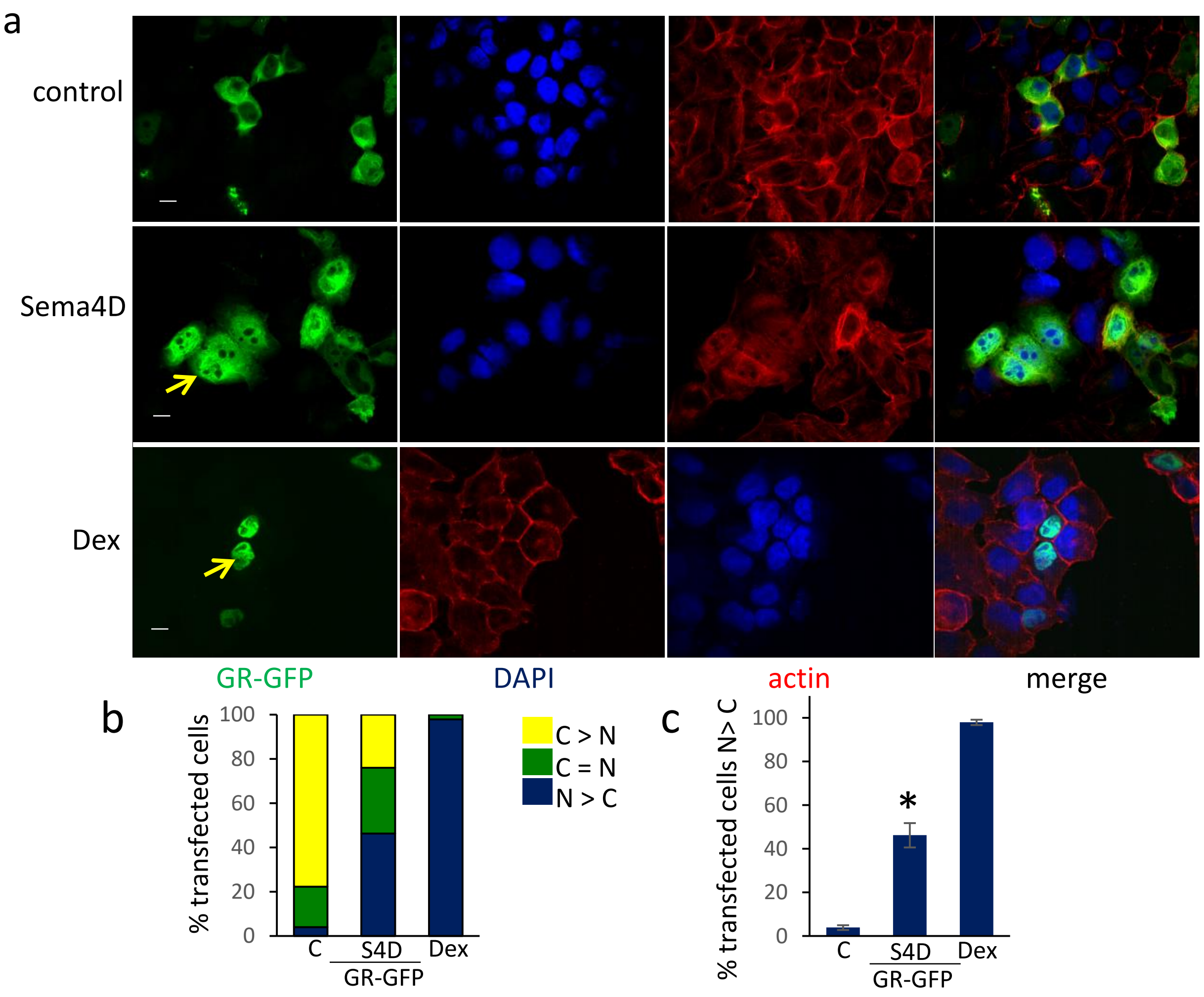

Supplementary Figure 3. Sema4D increases translocation of GR-GFP to the nucleus in Hela cells.
a).Representative images of Hela cells transfected with GR-GFP and treated with Sema4D, (2ug/ml), dexamethasone (10nM) or vehicle (control) for 60 mins. The cells were fixed and stained for actin (phalloidin-TRITC) and DNA (DAPI). Arrow denotes nuclear GR-GFP. Scale bar, 10um. b) Subcellular localisation of GR-GFP in transfected Hela cells. Following treatment with Sema4D, (2ug/ml), dexamethasone (10nM) or vehicle (control) for 60 mins, Hela cells transfected with GR-GFP were stained as in (a) and were scored blind and categorised into three groups: 1) nuclear GFP staining > cytoplasmic GFP staining (N>C), 2) nuclear and cytoplasmic GFP staining equal, (N=C), 3) cytoplasmic GFP staining> nuclear GFP staining (C>N) and the % of cells in each group scored (n=4). A total of 259+ cells were scored per treatment. c) Percentage of cells transfected with GR-GFP in which the intensity of nuclear GFP staining exceeded that of cytoplasmic staining (N>C) following treatment with Sema4D (2ug/ml), dexamethasone (10nM) or vehicle (control, C) for 60 mins (n=4). Error bars denote SEM, * $p<0.05$, Ttest)

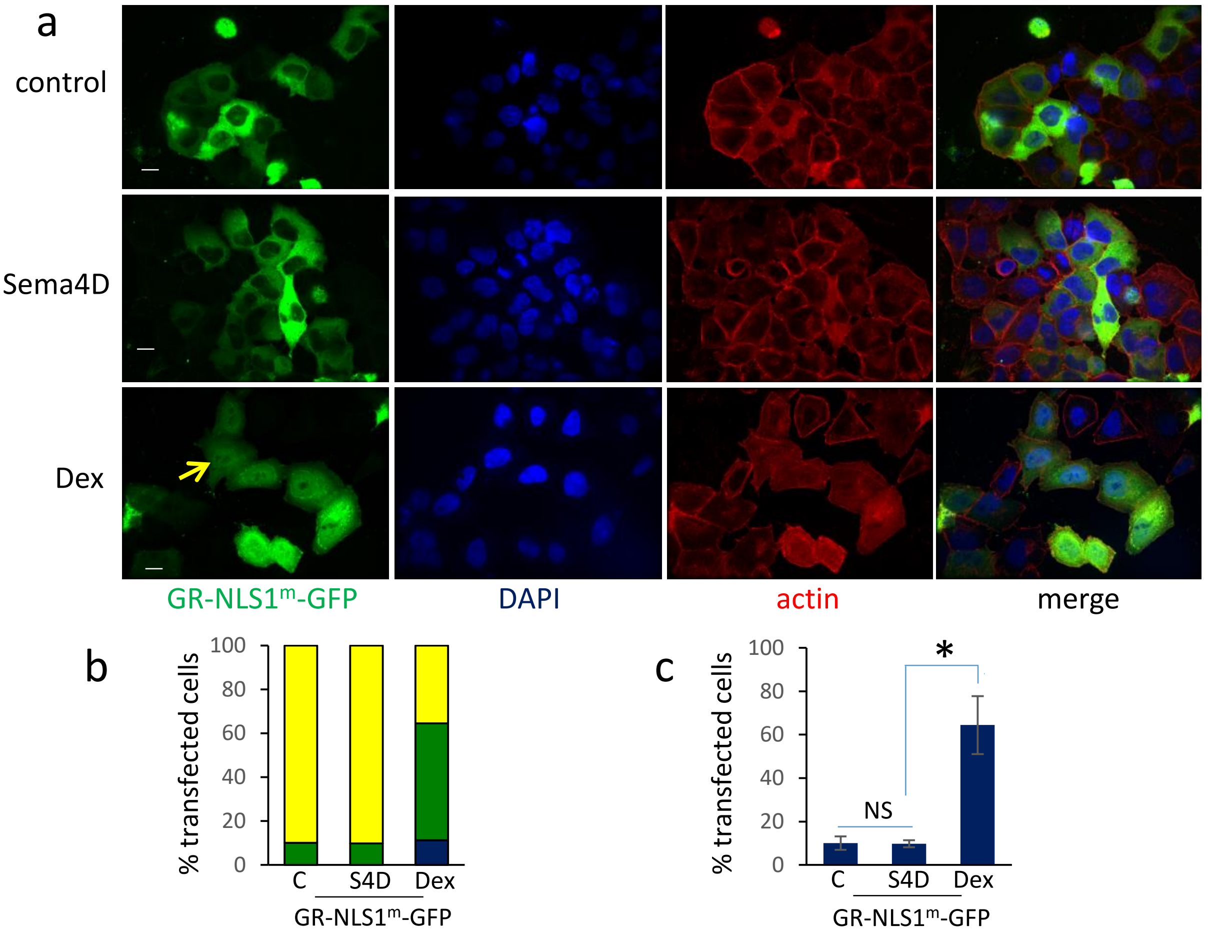

Supplementary Figure 4. Increase in Sema4D-mediated nuclear translocation of GR-GFP in Hela cells requires NLS1.

a). Representative images of Hela cells transfected with GR-NLS1^{m}-GFP and treated with Sema4D, (2ug/ml), dexamethasone (10nM) or vehicle (control) for 60 mins and stained as in supplementary figure 3. Arrow denotes nuclear GR-NLS1^{m}-GFP b) Subcellular localisation of GR-NLS1^{m}-GFP in transfected Hela cells. Following treatment with Sema4D, (2ug/ml), dexamethasone (10nM) or vehicle (control) for 60 mins, Hela cells transfected with GR-NLS1^{m}-GFP and stained and scored as in and stained as in supplementary figure 3. (n=4). A total of 182+ cells were scored per treatment. c)Percentage of cells transfected with GR-NLS1^{m}-GFP in which the intensity of nuclear GFP staining exceeded or was equal to that of cytoplasmic staining (N>C, N=C)) following treatment with Sema4D (2ug/ml), dexamethasone (10nM) or vehicle (control) for 60 mins (n=4). Error bars denote SEM, * p<0.05, NS= not significant, Ttest)

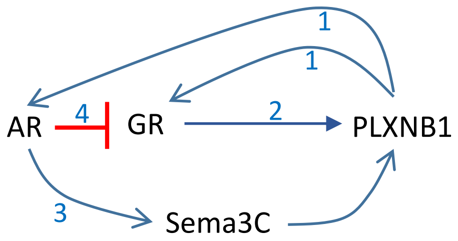

Supplementary Figure 5.
Diagram to show interplay between AR, GR and plexin signalling pathways
1).PlexinB1 stimulation promotes nuclear translocation and consequent activation of AR[29] and GR, 2). GR promotes PlexinB1 protein levels, 3). AR promotes Sema3C expression/PlexinB1 activation[38] 4).AR represses GR expression[3].
